# Morphological evidence of telocytes in human synovium

**DOI:** 10.1038/s41598-018-22067-5

**Published:** 2018-02-26

**Authors:** Irene Rosa, Mirca Marini, Daniele Guasti, Lidia Ibba-Manneschi, Mirko Manetti

**Affiliations:** Department of Experimental and Clinical Medicine, Section of Anatomy and Histology, University of Florence, Florence, Italy

## Abstract

A new cell type named telocyte (*i.e*. cell with distinctive prolongations called telopodes) has recently been identified in the stroma of various organs in humans. However, no study has yet reported the existence of telocytes in the synovial membrane of diarthrodial joints. This work was therefore undertaken to search for telocytes in the normal human synovium using transmission electron microscopy, immunohistochemistry and immunofluorescence. Ultrastructural analyses demonstrated the presence of numerous spindle-shaped telocytes in the whole synovial sublining layer. Synovial telocytes exhibited very long and thin moniliform telopodes and were particularly concentrated at the boundary between the lining and sublining layers and around blood vessels. Light microscopy confirmed the presence of CD34-positive telocytes in the aforementioned locations. Moreover, synovial telocytes coexpressed CD34 and platelet-derived growth factor receptor α. Double immunostaining further allowed to unequivocally differentiate synovial telocytes (CD34-positive/CD31-negative) from vascular endothelial cells (CD34-positive/CD31-positive). The *in vitro* examination of fibroblast-like synoviocyte primary cultures revealed the coexistence of different cell types, including CD34-positive telocytes projecting typical moniliform telopodes. In conclusion, our work provides the first evidence that telocytes do exist in the human synovium and lays the groundwork for future studies on synovial telocytes in a variety of degenerative and destructive joint diseases.

## Introduction

The term synovium (or synovial membrane) refers to a specialized soft connective tissue lining the inner surface of the fibrous capsule of diarthrodial joints, tendon sheaths and bursae that constitutes a critical provider of synovial fluid lubricant components and articular cartilage nutrients^1^. Hence, synovium is essential for joint homeostasis and synovial pathological changes are among the main drivers of a variety of degenerative and destructive joint diseases^1–4^.

The normal synovium is organized into two different layers: the synovial lining layer or intima that is in contact with the articular cavity and is constituted by compacted cells (one to three cells in thickness), and the underlying synovial sublining layer or subintima consisting of abundant collagenous extracellular matrix with dispersed fibroblast-like cells, macrophages, mast cells, autonomic nerve fibers, and blood and lymphatic vessels^1,4–6^. Unlike an epithelial layer, the synovial intima is devoid of a basement membrane with intimal cells being surrounded by a specialized extracellular matrix and forming sporadic and discrete regions of intercellular contacts^1,4–6^. The synovial lining layer comprises two morphologically different cell types: type A or macrophage-like synoviocytes that are bone marrow-derived phagocytic cells participating in clearance of debris from the joint cavity and serving as immune sentinels, and type B or fibroblast-like synoviocytes (also referred to as synovial fibroblasts), a kind of mesenchymal cells acting as major producers of synovial fluid molecular components, including hyaluronan and the glycoprotein lubricin^1,4–13^. At variance with the condensed synovial lining layer, the subintima consists of a loosely organized and highly vascularized connective tissue that constitutes a microanatomic support for the overlying synovial lining^1,4–7^. The sublining layer enables the transfer of either molecular or cellular components from the bloodstream to the lining and synovial fluid in the articular cavity^6^. In addition, the sublining contains immune competent cells for immunosurveillance and represents a potential reserve of fibroblast-like synoviocytes to ensure the maintenance of synovial lining integrity^6^. Furthermore, the adult human synovium seems to harbor mesenchymal progenitors that have the potential to differentiate into a wide variety of diarthrodial joint cell types, including chondroblasts, osteoblasts and adipocytes^6,14,15^. Although synovial fibroblast-like cells appear to be morphologically and functionally different between the lining and sublining layers^4^, there is little information concerning the possible existence of different fibroblast-like cell types in the synovial sublining compartment. Moreover, there are relatively few reported studies using transmission electron microscopy to determine the ultrastructural characteristics of synovial cells^7,8,16,17^.

Recently, a new stromal (interstitial) cell type with peculiar ultrastructural features called telocyte (TC) has been described in various organs of different species as cell with telopodes - very long cellular extensions with a moniliform silhouette featured by an alternation of thin segments (podomers) and dilated portions (podoms)^18–30^. Increasing evidence indicates that TCs can construct a complex three-dimensional network within the stromal compartment and release a variety of extracellular vesicles to regulate the functions of surrounding cells^18,31–35^. Indeed, TCs are suggested to play roles in tissue structural support, homeostasis maintenance, intercellular signaling, cell differentiation, immune surveillance, stem/progenitor cell guiding and nursing, and to participate in different tissue pathologies^18,19,31,35–47^. Especially, TCs are believed important in tissue regeneration, making them particularly attractive in the field of regenerative medicine^48,49^. In this way, it is of interest to investigate the possible presence of TCs in the synovial membrane. This work was therefore undertaken to search for TCs in the normal human synovium by means of transmission electron and light microscopy and lay the groundwork for future research on their behavior in synovial pathology.

## Results

**Figure 1:**
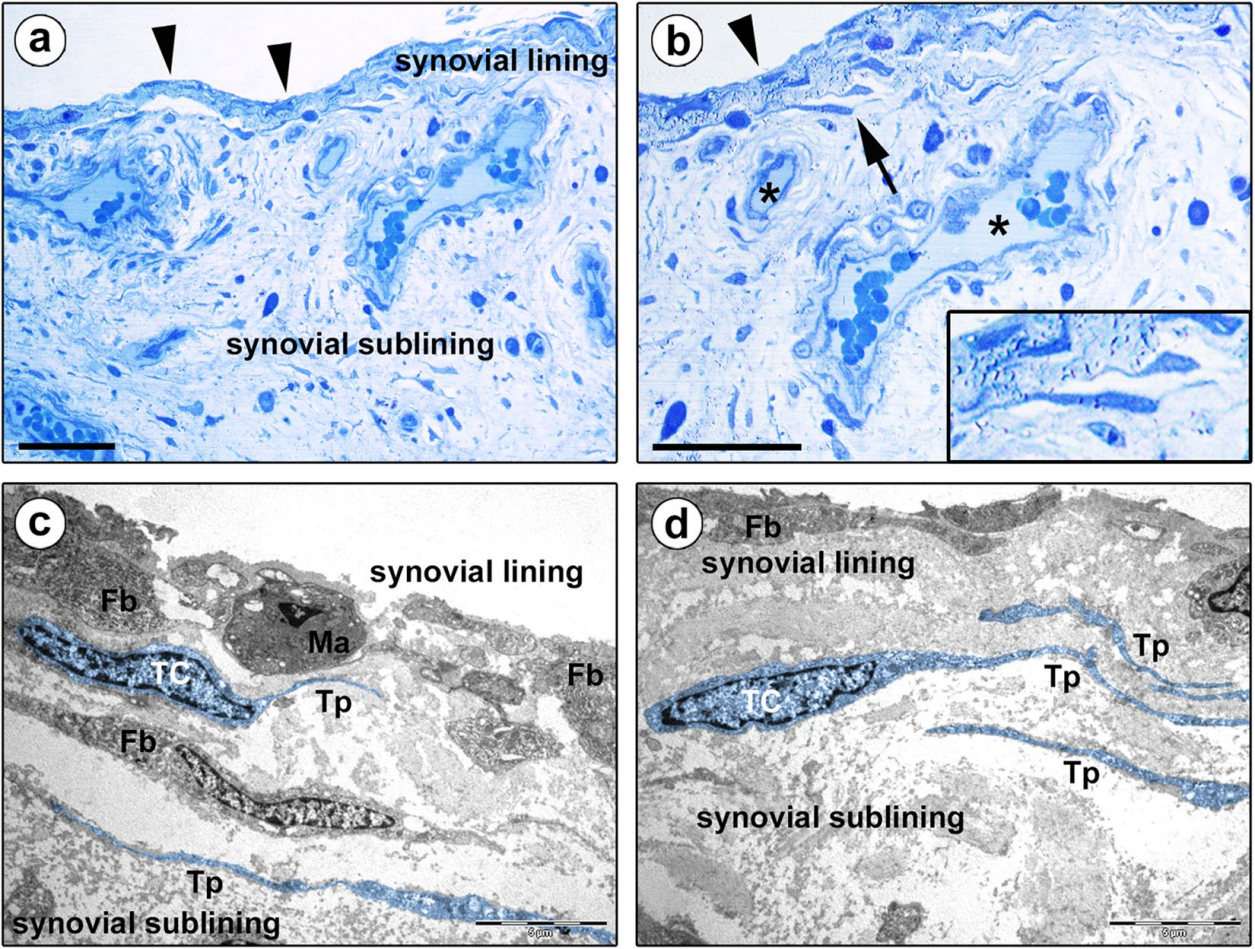
Morphology of human synovium (lining and sublining layers) under light and transmission electron microscopy. (**a**,**b**) Representative photomicrographs of toluidine blue-stained synovial semithin sections. Arrowheads in (**a**,**b**) point to the synovial lining layer, while asterisks in (**b**) denote blood vessels in the synovial sublining layer. (**b**) Stromal cells exhibiting a spindle-shaped or piriform cell body with a large nucleus and very small amount of cytoplasm, and long and thin cytoplasmic processes are observed at the boundary between the synovial lining and sublining layers (arrow; shown at higher magnification in the inset). (**c**,**d**) Representative transmission electron microscopy photomicrographs of synovial ultrathin sections stained with uranyl acetate and bismuth subnitrate solutions. Telocytes (TC) and telopodes (Tp) have been digitally colored in blue. The ultrastructural traits of telocytes are: i) a spindle-shaped or piriform cell body with a relatively large euchromatic nucleus surrounded by a thin cytoplasmic layer containing few mitochondria, scarce cisternae of endoplasmic reticulum and a small Golgi apparatus, and ii) the presence of telopodes, long cytoplasmic processes with a narrow emergence from the cell body and a moniliform silhouette characterized by the alternation of thin segments (podomers) and expanded parts (podoms). Telocytes and telopodes are present in the synovial sublining layer immediately beneath the type A/macrophage-like synoviocytes (Ma) and the type B/fibroblast-like synoviocytes (Fb) constituting the synovial lining (**c,d**). The synovial sublining contains either telopode-bearing telocytes or fibroblasts (Fb), these latter displaying an abundant cytoplasm rich in cisternae of rough endoplasmic reticulum, mitochondria and Golgi apparatus, and short and thick processes (**c**). Scale bar: 50 µm (**a**,**b**), 5 µm (**c**,**d**).

**Figure 2:**
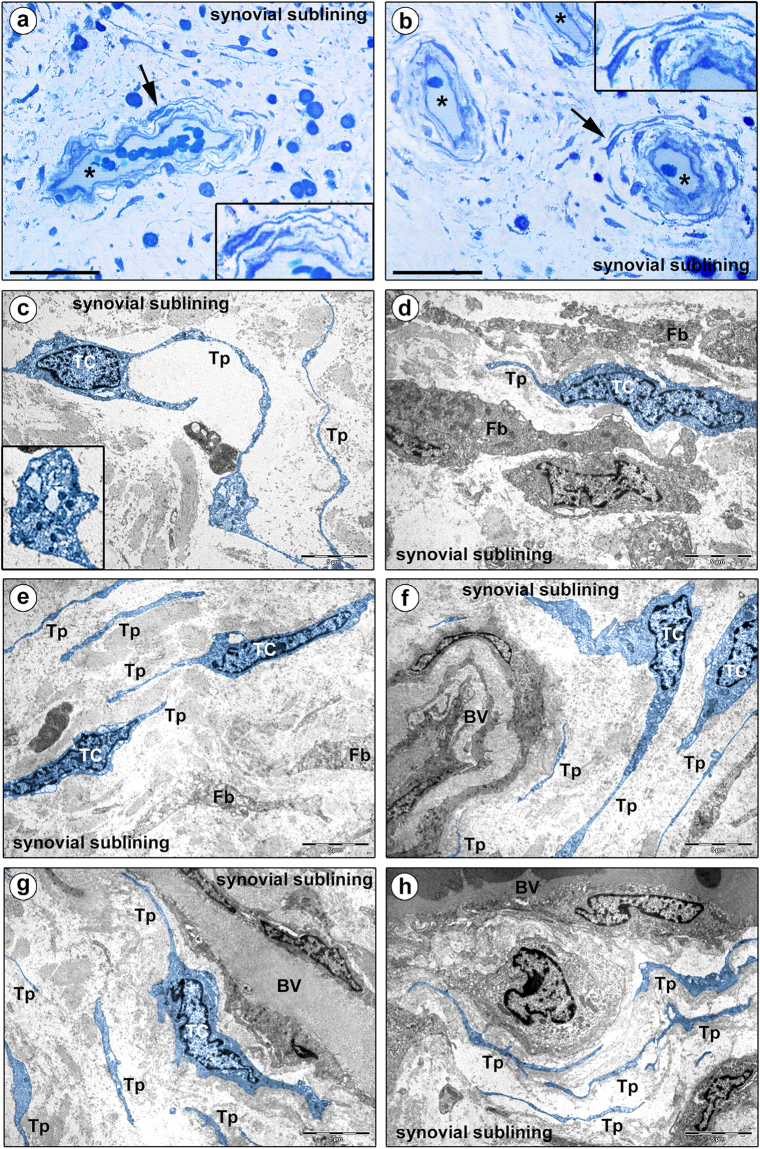
Morphology of human synovium (sublining layer) under light and transmission electron microscopy. (**a,b**) Representative photomicrographs of toluidine blue-stained synovial semithin sections. Asterisks denote sublining layer blood vessels. Stromal cells displaying a spindle-shaped or piriform cell body with a large nucleus and very small amount of cytoplasm, and long and thin moniliform cytoplasmic processes are present throughout the synovial sublining, particularly in perivascular locations (arrows; shown at higher magnification in the insets). (**c**–**h**) Representative transmission electron microscopy photomicrographs of synovial ultrathin sections stained with uranyl acetate and bismuth subnitrate solutions. Telocytes (TC) and telopodes (Tp) have been digitally colored in blue. Telocytes are ultrastructurally characterized by i) a small cell body mostly occupied by a relatively large nucleus surrounded by scarce cytoplasm containing few mitochondria and cisternae of endoplasmic reticulum and a small Golgi apparatus, and ii) thin moniliform processes (telopodes) with narrow emergence from the cell body and a moniliform aspect (alternating thin segments/podomers and dilated portions/podoms). The nucleus is indented, showing patches of heterochromatin near the nuclear membrane. Podoms accommodate some organelles, such as rough endoplasmic reticulum and mitochondria (**c**, inset). The ultrastructural traits of telocytes make them easily distinguishable from neighboring fibroblasts (Fb), which exhibit a large body rich in cisternae of rough endoplasmic reticulum and mitochondria, a large Golgi apparatus and short and thick processes (**d**). Telocytes and telopodes can be observed throughout the whole synovial sublining layer, either in neutral positions surrounded by abundant collagenous extracellular matrix (**c,e**), or topographically closely related to fibroblasts (**d**) and blood vessels (BV) (**f**–**h**). Note the presence of telopodes delimiting the basal lamina of microvessels (**f**–**h**). Scale bar: 50 µm (**a,b**), 5 µm (**c**–**h**).

Normal human knee synovium semithin sections were subjected to toluidine blue staining to examine the general morphological features of the synovial lining and sublining layers, followed by ultrastructural analysis of ultrathin sections to investigate the existence of TCs and to determine their localization according to the definition by Cretoiu and Popescu^18^ (Figs 1a–d and 2a–h).

Cells with long and thin cytoplasmic processes were observed in toluidine blue-stained synovial semithin sections (Figs 1a,b and 2a,b). They exhibited a spindle-shaped or piriform cell body containing a large nucleus and very small amount of cytoplasm and were widely distributed in the synovial subintima, being particularly concentrated at the boundary between the lining and sublining layers and around blood vessels (Figs 1b and 2a,b, insets). However, since the morphology of those stromal cells was difficult to characterize precisely under conventional light microscopy, we next investigated in depth their ultrastructural traits.

Based on the peculiar ultrastructural features described in the literature^18,19^, it was possible to unequivocally distinguish synovial TCs from other stromal cells by transmission electron microscopy. Synovial TCs displayed a euchromatic nucleus often slightly indented, showing patches of heterochromatin particularly near the nuclear membrane, and relatively large in relation to the small content of cytoplasm in the cell body (Figs 1c,d and 2c–g). Their somatic body presented a thin cytoplasmic layer containing few mitochondria, scarce cisternae of endoplasmic reticulum and a small Golgi apparatus (Figs 1c,d and 2c–g). The most distinctive ultrastructural feature of TCs was the presence of telopodes, long cytoplasmic processes (up to ~70 µm; mean ± SD length, 29.3 ± 15.6 µm) with a narrow emergence from the cellular body and a moniliform aspect due to the alternation of extremely slender segments (podomers; mean ± SD thickness, 88.3 ± 57.4 nm) and expanded parts (podoms; mean ± SD thickness, 382.6 ± 118.2 nm) containing some organelles, such as rough endoplasmic reticulum and mitochondria (Figs 1c,d and 2c–f, inset). These morphological characteristics made TCs rather different from neighboring fibroblasts, which were instead characterized by a large body rich in cisternae of rough endoplasmic reticulum and mitochondria, a large Golgi apparatus and short and thick processes (Figs 1c and 2d).

As displayed in Fig. 1c,d, transmission electron microscopy showed the presence of numerous TCs and telopodes immediately beneath the type A/macrophage-like and type B/fibroblast-like synoviocytes constituting the synovial lining layer. Moreover, numerous TCs and telopodes were identified in the deeper synovial sublining layer either in neutral positions surrounded by abundant collagenous extracellular matrix, or topographically closely related to fibroblasts and microvessels (Fig. 2c–h). In particular, telopodes were observed frequently to delimit the basal lamina of small blood vessels (Fig. 2f–h).

**Figure 3:**
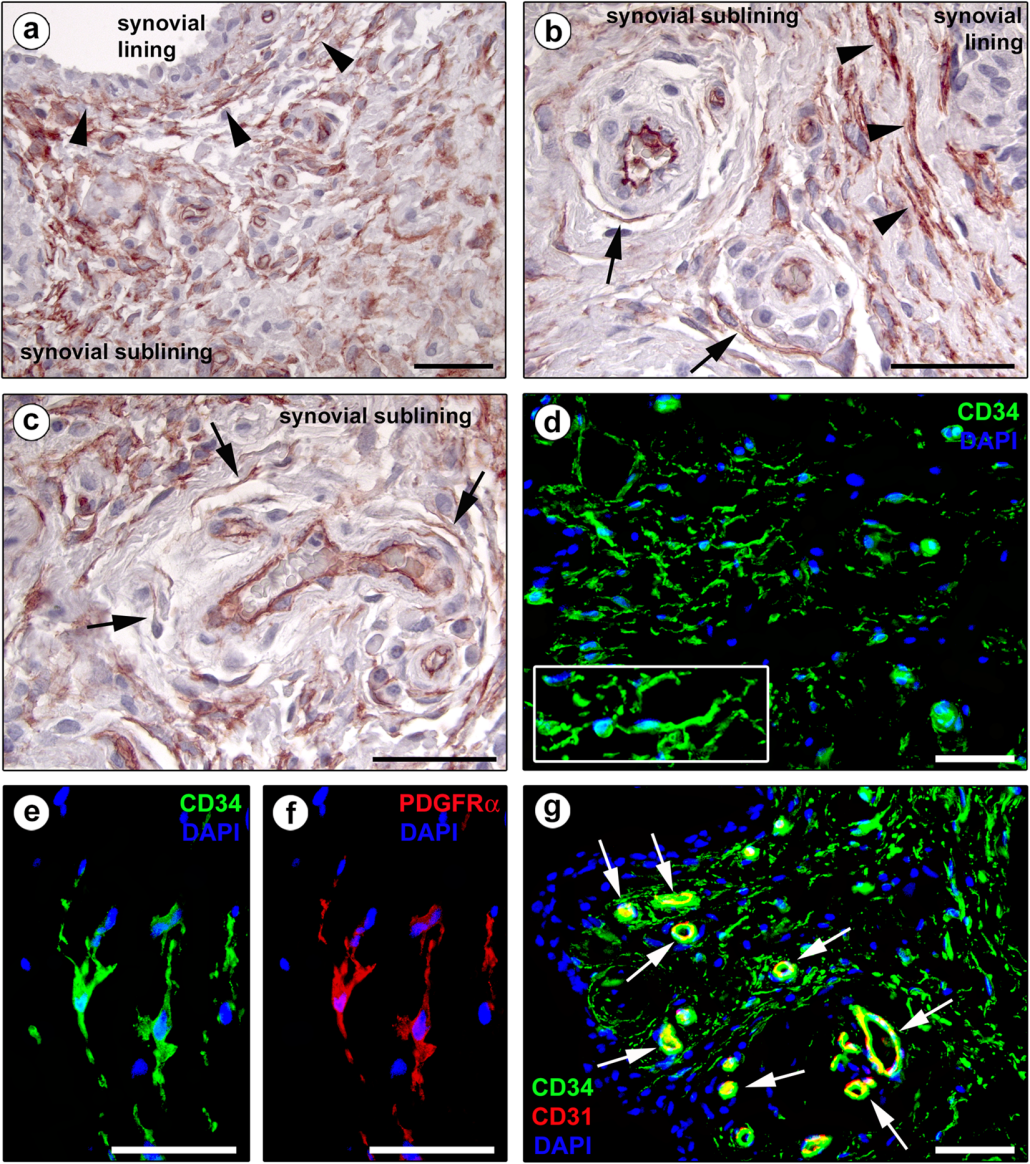
Representative light and fluorescence microscopy photomicrographs of human synovial sections. (**a**–**c**) CD34 immunoperoxidase-based immunohistochemistry with hematoxylin counterstain. (**d**) Immunofluorescence labeling for CD34 (green) with 4′,6-diamidino-2-phenylindole (DAPI; blue) counterstain for nuclei. CD34-positive spindle-shaped cells (telocytes) with long cytoplasmic processes (telopodes) are distributed throughout the whole synovial sublining layer (**a**–**d**). Note the presence of numerous CD34-positive stromal cells arranged in multiple parallel rows at the synovial lining-sublining interface (arrowheads in **a,b**) and surrounding sublining microvessels (arrows in **b,c**). CD34 immunopositivity is observed also in vascular endothelial cells. At higher magnification, the processes of CD34-positive stromal cells exhibit a moniliform silhouette (**d**, inset). (**e,f**) Double immunofluorescence labeling for CD34 (green) and PDGFRα (red) with DAPI (blue) counterstain for nuclei. Telocytes are double positive for CD34 and PDGFRα. (**g**) Double immunofluorescence labeling for CD34 (green) and CD31 (red) with DAPI (blue) counterstain for nuclei. Telocytes are CD34-positive/CD31-negative, while vascular endothelial cells are CD34-positive/CD31-positive (arrows). Scale bar: 50 µm (**a**–**g**).

According to previously published studies^18,43,44,50^, TCs were further identified by CD34 immunostaining, using both immunoperoxidase-based immunohistochemistry and immunofluorescence. In accordance with the TC locations identified by transmission electron microscopy, CD34-positive spindle-shaped cells with long and moniliform processes and often chained in networks were seen throughout the whole synovial sublining layer (Fig. 3a–d). As shown in Fig. 3a,b, these CD34-expressing stromal cells were arranged in multiple parallel rows at the synovial lining-sublining interface. In the synovial sublining, their networks surrounded microvessels (Fig. 3b,c). As expected, CD34 expression was also found in vascular endothelial cells. In addition, synovial TCs were double positive for CD34 and platelet-derived growth factor receptor α (PDGFRα) (Fig. 3e,f), which is presently considered one of the best marker combinations to identify TCs by light microscopy^18,44,47,51,52^. Double immunofluorescence further allowed to unequivocally differentiate synovial TCs (CD34-positive/CD31-negative) from vascular endothelial cells (CD34-positive/CD31-positive) (Fig. 3g).

**Figure 4:**
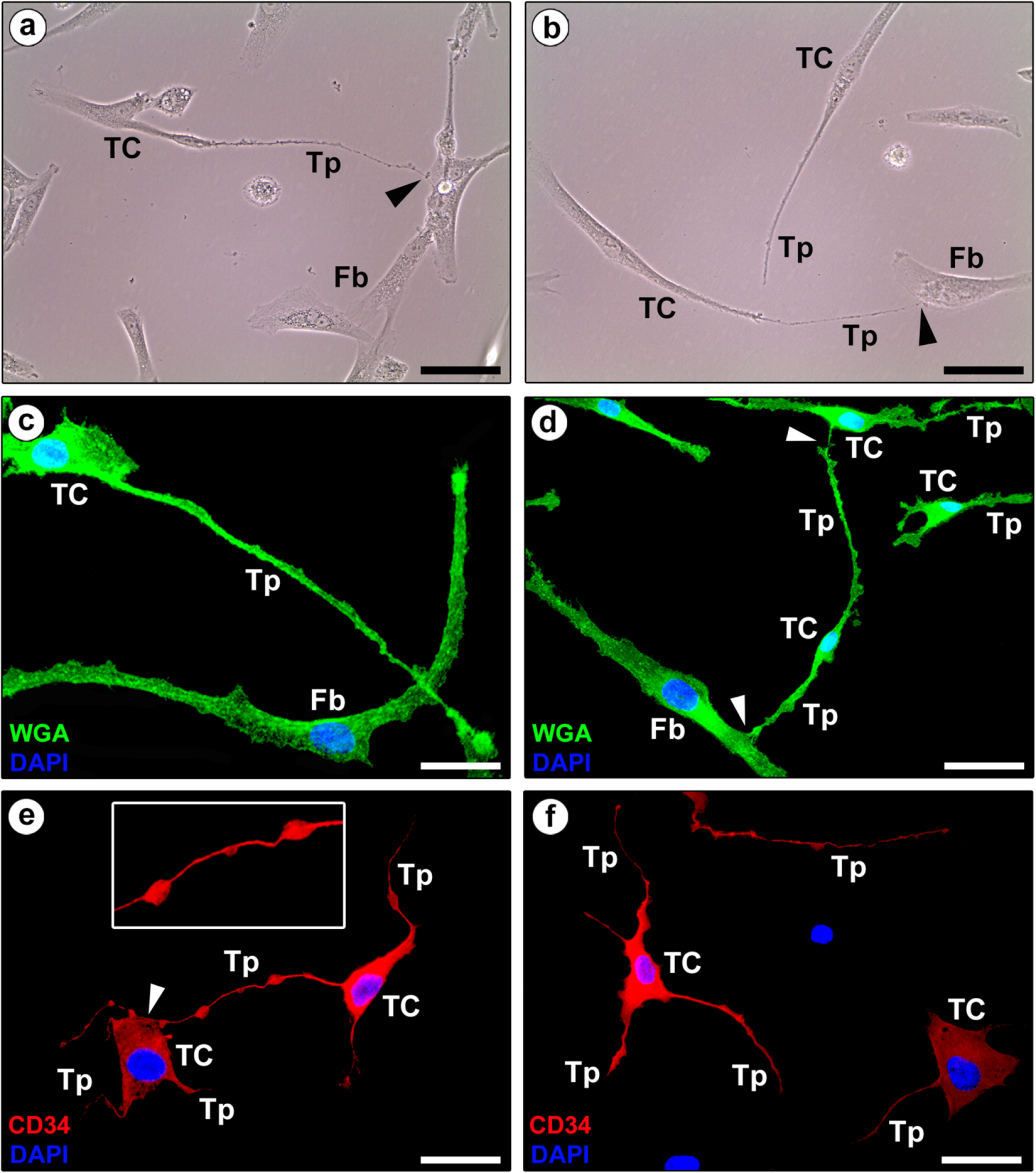
Representative phase-contrast and fluorescence microscopy photomicrographs of human fibroblast-like synoviocyte primary cultures. (**a,b**) Phase-contrast microscopy. (**c,d**) Wheat Germ Agglutinin (WGA; green) immunofluorescence labeling with 4′,6-diamidino-2-phenylindole (DAPI; blue) counterstain for nuclei. (**a**–**d**) Note the presence of either fibroblasts (Fb) or telocytes (TC), the latter being characterized by a small cell body and very long and thin moniliform processes (telopodes, Tp) often establishing intercellular contacts with fibroblasts or other telocytes (arrowheads). (**e,f**) Immunofluorescence labeling for CD34 (red) with DAPI (blue) counterstain for nuclei. CD34-positive cells projecting very long and thin moniliform cytoplasmic processes (telopodes, Tp) are unequivocally identifiable as telocytes (TC). The inset in (**e**) shows a higher magnification of a typical telopode formed by an alternation of thin segments (podomers) and expanded parts (podoms). Note the telopode of a telocyte contacting the cell body of another telocyte (arrowhead in **e**). Scale bar: 50 µm (**a**–**f**).

Finally, the *in vitro* examination of normal human fibroblast-like synoviocyte primary cultures revealed the coexistence of different cell types, including TCs displaying a small cell body and very long and thin moniliform extensions often establishing intercellular contacts with fibroblasts or other TCs (Fig. 4a–d). The presence of synovial TCs in primary cultures was further confirmed by fluorescence immunocytochemistry, which demonstrated numerous CD34-positive cells projecting typical moniliform telopodes (Fig. 4e,f). In comparison with synovial ultrathin sections for transmission electron microscopy which contained only fragments of telopodes, cell culture had the advantage to show almost the entire telopodes revealing lengths of hundreds of micrometers.

## Discussion

In the last few years numerous data were reported showing the existence of a novel stromal cell type, namely TCs (formerly referred to as interstitial Cajal-like cells), located in the connective tissue of a variety of cavitary and non-cavitary organs in humans, rodents and other species^18–30,33,43,47,50,51,53,54^.

As far as the musculoskeletal (locomotor) system is concerned, the existence of TCs has recently been demonstrated in skeletal muscles, neuromuscular spindles, fascial structures and temporomandibular joint disc^24,55–59^. In this context, the findings of our study provide the first evidence that TCs are part of the microscopic anatomical structure of synovium, a thin connective tissue membrane that covers intraarticular surfaces of the fibrous joint capsule and serves as an important source of nutrients and lubricants for diarthrodial joints. Indeed, the results reported here show unequivocally that the stromal cells we are describing in the human synovial sublining layer fulfill the criteria for the positive diagnosis of TCs, including key ultrastructural features - a small cell body projecting very long distinctive telopodes made by an alternation of podomers and podoms, and positive immunoreactions for CD34 and PDGFRα^18^. Although transmission electron microscopy remains the gold standard for tissue identification of TCs, compelling evidence indicates that CD34-positive stromal cells are indeed TCs^18,19,50^. Of note, cells referred to as CD34-positive stromal fibroblastic/fibrocytic cells have previously been noticed in the underlying tissue of synovia^50,60^.

As already documented in the stromal compartment of many human organs^43,44,47,50,51^, we found that the extremely long processes of CD34-positive TCs form interstitial networks distributed throughout the whole connective tissue constituting the human synovial subintima. Moreover, transmission electron microscopy clearly demonstrated the coexistence of ultrastructurally distinct TCs and fibroblasts in the sublining layer. On the contrary, either ultrastructural or immunohistochemical analyses showed that TCs are absent from the synovial lining layer, which appears to be populated exclusively by the so-called type A/macrophage-like and type B/fibroblast-like synoviocytes. Besides the aforementioned *ex vivo* tissue findings, the existence of peculiar synovial TCs was confirmed *in vitro* through an investigation of primary cultures of human fibroblast-like synovial cells.

Owing to the descriptive study design, our data do not provide direct information about the presumptive functions of synovial TCs. However, according to the different roles that have been ascribed to the TCs in other organs^18,19,31,35,42,49^, the three-dimensional network formed by telopodes within the sublining layer might be endowed with mechanical support, intercellular communication and regulatory properties making a substantial contribution to the maintenance of synovial homeostasis. Noteworthy, the location of numerous TCs and telopodes at the boundary between the synovial lining and sublining layers and in close vicinity of blood microvessels supports this assumption. Indeed, by their long-distance spreading and interconnecting processes synovial TCs seem to behave primarily as compartmentalizing cells, and likely they regulate the transfer of both molecular and cellular components from sublining blood vessels to the lining layer and synovial fluid in the joint cavity. As reported in cardiac and prostate gland morphogenesis^61,62^, such compartmentalizing function of TCs might even help in driving the correct synovial tissue organization during diarthrodial joint development, though this will need to be testified by further research. Interestingly, a proportion of the synovial fibroblast-like cells are believed to act as adult stem cells that may contribute to joint tissue renewal and repair^14,15^, and a large body of evidence suggests that TCs could indeed fulfill this function^35,44,49,50,63^. Further, one can speculate that synovial TCs likewise TCs in other organs, such as those in the intestinal lamina propria or in the skin, might be involved in immunomodulatory mechanisms and immune surveillance, acting like ‘local data suppliers’ for inflammatory and immune responses^18,22,42^. In addition, TCs have been suggested to participate in a variety of processes, such as regulation of angiogenesis, inhibition of oxidative stress and prevention of cellular aging^42^. Considering that synovial inflammation and blood vessel overgrowth are predominant features and causes of joint degeneration in diseases as diverse as rheumatoid, psoriatic, juvenile and idiopathic arthritis, lupus, gout and Lyme disease^2^, future research employing diseased tissues has the great potential to help in clarifying the role(s) of synovial TCs.

## Conclusions

In summary, our work demonstrates that TCs are a previously neglected cellular entity of the microscopic anatomical structure of human synovium and lays the foundations for upcoming studies on synovial TCs in a variety of degenerative and destructive joint diseases. To understand synovial TC role(s), further work is clearly warranted using dynamic methods either *in vitro* or *in vivo*. Unraveling the possible functions of synovial TCs might indeed provide new clues for their potential therapeutic applications in joint regenerative medicine.

## Methods

### Human synovial specimens

The study was entirely carried out on epoxy resin- and paraffin-embedded normal human synovial tissue samples collected at the archives of the Section of Anatomy and Histology, Department of Experimental and Clinical Medicine, University of Florence. Archival samples of synovium were obtained from 6 healthy adult male subjects who had undergone post-traumatic knee surgical intervention and had signed a written informed consent form. The study was carried out in accordance with the Declaration of Helsinki and approved by the Institutional Review Board at the Careggi University Hospital, Florence, Italy. Immediately after sampling, synovial specimens were divided into small pieces and processed for transmission electron and light microscopy.

### Transmission electron microscopy

Transmission electron microscopy was carried out according to previously published protocols^44^. Small human synovial specimens were fixed in 4% cacodylate-buffered glutaraldehyde (pH 7.4) at room temperature, rinsed in a cacodylate-buffered solution supplemented with sucrose, post-fixed in 1% OsO_4_ (Electron Microscopy Sciences, Foster City, CA, USA), dehydrated with graded alcohol series, immersed in propylene oxide and embedded in Epon 812 resin. Semithin sections (2 μm thick) were obtained with a RMC MT-X ultramicrotome (EMME3, Milan, Italy) and stained with a toluidine blue solution in 0.1 M borate buffer, and then examined and photographed under a light microscope. Ultrathin sections (~70 nm thick) of the selected areas were cut with the same ultramicrotome using a diamond knife and stained with an alcoholic solution of uranyl acetate, followed by an alkaline bismuth subnitrate solution. Ultrathin sections were observed and photographed under a JEOL JEM-1010 electron microscope (Jeol, Tokyo, Japan) equipped with a MegaView III high-resolution digital camera and imaging software (Jeol). Transmission electron microscopy images were digitally colored using Adobe Photoshop CS6 software (Adobe Systems, San Jose, CA, USA) in order to highlight TCs and telopodes. Telopode length and width were measured on at least 20 randomly selected structures per synovial sample. Measurements were performed on the acquired photomicrographs employing the ImageJ software (NIH, Bethesda, MD, USA) as described elsewhere^44^.

### Immunoperoxidase-based immunohistochemistry

Immunohistochemistry was carried out on formalin-fixed and paraffin-embedded synovial tissue blocks following previously established protocols^44,47,64^. Synovial sections (5 µm thick) were deparaffinized, boiled for 10 minutes in sodium citrate buffer (10 mM, pH 6.0) for antigen unmasking and then treated with 3% H_2_O_2_ solution for 15 minutes at room temperature to block endogenous peroxidases. After blockade of non-specific antibody binding by incubation with Ultra V block (UltraVision Large Volume Detection System Anti-Polyvalent, HRP, catalog number TP-125-HL; Lab Vision, Fremont, CA, USA) for 10 minutes at room temperature, tissue sections were incubated overnight at 4 °C with a mouse monoclonal anti-human CD34 antibody (1:50 dilution; clone QBEnd-10, catalog number M7165; Dako, Glostrup, Denmark). The subsequent day, tissue slides were extensively washed in phosphate-buffered saline (PBS) and incubated with biotinylated secondary antibodies (Lab Vision) for 10 minutes at room temperature according to the manufacturer’s instructions. Tissue sections were then washed in PBS and treated with streptavidin peroxidase (Lab Vision) for 10 minutes at room temperature followed by chromogenic development of immunoreactivity using 3-amino-9-ethylcarbazole (AEC kit, catalog number TA-125-SA; Lab Vision). Synovial sections were finally counterstained with hematoxylin (Bio-Optica, Milan, Italy) and observed under a Leica DM4000 B microscope (Leica Microsystems, Mannheim, Germany). Sections exposed to isotype- and concentration-matched irrelevant mouse IgG (Sigma-Aldrich, St. Louis, MO, USA) were run in parallel as negative controls. Light microscopy images were captured with a Leica DFC310 FX 1.4-megapixel digital color camera equipped with the Leica software application suite LAS V3.8 (Leica Microsystems).

### Immunofluorescence staining of synovial tissue sections

Single and double immunofluorescence combining anti-CD34 either with anti-PDGFRα or anti-CD31/platelet-endothelial cell adhesion molecule-1 (PECAM-1) antibodies was performed according to previously published studies^43,44,46,47^. Synovial tissue sections (5 µm thick) were deparaffinized, rehydrated and subjected to antigen retrieval by boiling in sodium citrate buffer (10 mM, pH 6.0). Slides were rinsed in PBS, exposed to a glycine solution for 10 minutes to quench autofluorescence, and subsequently blocked for 1 hour at room temperature with 1% bovine serum albumin (BSA) in PBS. Tissue sections were then incubated overnight at 4 °C with primary antibodies diluted in PBS with 1% BSA. The primary antibodies used were mouse monoclonal anti-human CD34 (1:50 dilution; catalog number M7165; Dako), goat polyclonal anti-human PDGFRα (1:100 dilution; catalog number AF-307-NA; R&D Systems, Minneapolis, MN, USA) and rabbit polyclonal anti-human CD31/PECAM-1 (1:50 dilution; catalog number ab28364; Abcam, Cambridge, UK). After three washes in PBS, the slides were incubated for 45 minutes at room temperature in the dark with the following secondary antibodies: Alexa Fluor-488-conjugated donkey anti-mouse IgG, Alexa Fluor-568-conjugated donkey anti-goat IgG or Rhodamine Red-X-conjugated goat anti-rabbit IgG (Invitrogen, San Diego, CA, USA), all diluted 1:200 in PBS with 1% BSA. Double immunofluorescence staining was carried out by mixing mouse and rabbit or goat primary antibodies followed by a mixture of fluorochrome-conjugated IgG. Irrelevant isotype- and concentration-matched mouse, rabbit and goat IgG (Sigma-Aldrich) served as negative controls. Control experiments omitting primary antibodies were performed in parallel in order to exclude cross-reactivity of secondary antibodies. Nuclei were counterstained with 4′,6-diamidino-2-phenylindole (DAPI; Chemicon International, Temecula, CA, USA). Synovial sections were finally mounted with an antifade aqueous mounting medium, observed under a Leica DM4000 B microscope with fully automated fluorescence axes, and photographed with a Leica DFC310 FX 1.4-megapixel digital color camera equipped with the Leica software application suite LAS V3.8 (Leica Microsystems).

### Cell culture and fluorescence microscopy

Human fibroblast-like synoviocytes (HFLS, adult) were purchased from Cell Applications Inc. (San Diego, CA, USA) and cultured in RPMI 1640 medium (Invitrogen) supplemented with 15% heat-inactivated fetal bovine serum, penicillin-streptomycin and gentamycin in a humidified atmosphere of 5% CO_2_ at 37 °C. At confluence, cells were trypsinized and recultured in medium. Cells between passages 4 and 6 were subjected to experimental procedures. Phase-contrast images were obtained under a Leica inverted microscope (Leica Microsystems) to assess cell morphology. Fluorescence immunocytochemistry was carried out as reported elsewhere^64^. Briefly, cells were grown onto glass coverslips, fixed with 3.7% buffered paraformaldehyde and then permeabilized with 0.1% Triton X-100 in PBS. Cells were rinsed in PBS, blocked with 1% BSA in PBS for 1 hour at room temperature and incubated overnight in a humidified chamber at 4 °C with mouse monoclonal anti-human CD34 (clone QBEnd-10, catalog number M7165; Dako) at 1:20 dilution in 1% BSA in PBS. The day after, slides were incubated for 45 minutes at room temperature in the dark with Rhodamine Red-X-conjugated anti-mouse IgG (1:200 dilution; Invitrogen). Negative controls were performed using irrelevant isotype- and concentration-matched mouse IgG (Sigma-Aldrich). To stain plasma membranes of cells, paraformaldehyde-fixed slides were rinsed in PBS and incubated for 10 minutes at room temperature in the dark with Alexa Fluor-488-conjugated Wheat Germ Agglutinin (WGA; Thermo Fisher Scientific, Waltham, MA, USA) at 1:200 dilution. Nuclei were counterstained with DAPI. Immunostained cells were examined with a Leica DM4000 B microscope and fluorescence images were captured with a Leica DFC310 FX 1.4-megapixel digital color camera (Leica Microsystems).

### Data availability

All relevant data are within the paper.
